# Supplementary material for: Somnotate: A probabilistic sleep stage classifier for studying vigilance state transitions
Source: PLoS Comput Biol. 2024 Jan 17;20(1):e1011793. doi: 10.1371/journal.pcbi.1011793 (PMC10824458; doi:10.1371/journal.pcbi.1011793)
Supplement: S1 Appendix — (DOCX) [file pcbi.1011793.s001.docx]

Somnotate: A probabilistic sleep stage classifier for studying vigilance state transitions

Paul J. N. Brodersen^1^, Hannah Alfonsa^1^, Lukas B. Krone^2^, Cristina Blanco-Duque^2^, Angus S. Fisk^3^, Sarah J. Flaherty^2^, Mathilde C. C. Guillaumin^3,4,5^, Yi-Ge Huang^2^, Martin C. Kahn^2^, Laura E. McKillop^2^, Linus Milinski^2^, Lewis Taylor^3^, Christopher W. Thomas^2^, Tomoko Yamagata^3^, Russell G. Foster^4^, Vladyslav V. Vyazovskiy^2^, Colin J. Akerman^1^

***1*** Department of Pharmacology, University of Oxford; Mansfield Road, Oxford, UK.
***2*** Department of Physiology, Anatomy and Genetics, University of Oxford; Parks Road, Oxford, UK.

***3*** Nuffield Department of Clinical Neurosciences, University of Oxford; Level 6, West Wing, John Radcliffe Hospital, Oxford, UK.

***4*** Sleep and Circadian Neuroscience Institute, University of Oxford; Parks Road, Oxford, UK.

***5*** Institute for Neuroscience, Department of Health Sciences and Technology, ETH Zurich; Schorenstrasse 16, Schwerzenbach, Switzerland.

# Supplementary Information**:** Appendix S1

## Unbiased and precise assessment of automated and manual sleep annotation

1. The performance of sleep stage classifiers is typically measured by computing their agreement with two independent manual annotations. Performance is evaluated as the average agreement of the automated annotation with each of the two manual annotations, and this average is then compared to the level of agreement between the two manual annotations. This subtle difference in how manual and automated annotations are compared can lead to systematic biases in favour of the automated annotation. For example, assume that one manual annotation is perfectly accurate but the other manual annotation misclassifies half of the data. The inter-rater agreement between manual annotations is calculated as 1 * 0.5 = 0.5. Now assume that the automated annotation has exactly the average accuracy of the two manual annotations, i.e. 0.75. The average agreement with the two manual annotations will be (1 * 0.75 + 0.5 * 0.75) / 2 = 0.5625. In other words, the automated annotation will appear to be more than 10% better than the manual annotations, even though its accuracy was exactly average. Conversely, to achieve an average agreement score of 0.5, the automated annotation would only need to have an accuracy of 0.667, i.e. it could be 10% less accurate than the mean manual accuracy, while still achieving the same agreement between manual annotations.
2. For these reasons, we were keen to compare automated annotations to a majority-vote consensus derived from multiple independent manual annotations. We asked ten experienced sleep researchers (**S1 Table**) to annotate awake, NREM, and REM states from the same 12-hour data set based on simultaneous recordings of an anterior EEG, posterior EEG, and EMG in a freely behaving mouse (Materials and methods). This enabled us to generate consensus annotations based on multiple independent manual annotations (**S5A-C Fig**). First, we assessed the accuracy of each annotation against the consensus of the other nine annotations. This revealed that although the overall accuracy of the annotations was high, individual annotations varied in terms of how closely they matched the consensus of the other annotations (**S5C-D Fig**). This variance would cause systematic bias if one was to rely upon the level of agreement between just two manual annotations (see above). We were also keen to assess how precisely the agreement between any two annotators is able to capture the mean accuracy of both manual annotations. We therefore compared the inter-rater agreement for each pair of annotations to the mean of their accuracies based on the majority-vote consensus of the remaining eight annotations (serving as a proxy for ground truth). Whilst there was a statistically significant linear relationship between inter-rater agreement and the mean accuracy of the two annotations, the relationship was weak (R^2^ = 0.25; **S5E Fig**).
3. To assess the quality of manual and automated annotations in an unbiased and more precise way, we compared the annotations to the consensus of multiple independent manual annotations. This comparison is unbiased as both the manual and automated annotations are assessed in exactly the same way. We confirmed that it is also a more precise measure, as the spread of performance estimates of manual annotations was smaller when using the consensus of three independent manual annotations to assess the accuracy of a fourth annotation, than when using a single other annotation as a point of reference (p < 0.01, Wilcoxon signed rank test; **S5F Fig**). Finally, to estimate the minimum number of manual annotations required to achieve a high quality consensus sequence, we determined the consensus sequence of five annotations by majority-vote. Using either one, three or all five of the remaining unused annotations, we constructed a second consensus sequence, and computed the agreement between the two and then repeated this process for all possible combinations. On average, any individual manual annotation matched a consensus of five sequences for 92.5% ± 1.3% of the data (mean ± standard deviation), whereas a consensus of three annotations already significantly increased the agreement by 2.2% ± 1.5% (agreement 94.7% ± 0.8%; p < 0.01, Mann-Whitney rank test; **S5G Fig**). There was a significant but more modest improvement of 0.5% ± 1.1%, when the number of manual annotations was increased to five (agreement 95.3% ± 0.7%; p < 0.01, Mann-Whitney rank test). Another widely used measure of inter-rater agreement is Cohen’s kappa, which accounts for the possibility of agreements occurring due to chance. When we repeated the analyses using this performance measure, we obtained analogous results (**S6 Fig)**.
4. In summary, a consensus derived from multiple independent manual annotations provides a less biased and more precise framework for assessing the quality of manual and automated annotations under comparable conditions. Based on these observations, we generated a larger test data set of six 24-hour EEG and EMG recordings (i.e. 144 hours total), which were independently scored by at least four experienced sleep researchers. This allowed us to compute the accuracy of manual and automated annotations using the majority-vote consensus of at least three other manual annotations for that recording. The recordings, individual manual annotations, and automated annotations are made freely available in standard formats (see Data Availability Statement).
