# Supplementary material for: Somnotate: A probabilistic sleep stage classifier for studying vigilance state transitions
Source: PLoS Comput Biol. 2024 Jan 17;20(1):e1011793. doi: 10.1371/journal.pcbi.1011793 (PMC10824458; doi:10.1371/journal.pcbi.1011793)
Supplement: S1 Table — All authors who provided manual annotations reported their task-relevant experience in years, plus the approximate number of hours that they had previously manually annotated. (DOCX) [file pcbi.1011793.s002.docx]

| **Annotator** | **Experience (Years)** | **Total data annotated (Hours)** |
| --- | --- | --- |
| VVV | 22 | 10000 |
| TY | 6 | 3840 |
| LEM | 7 | 2400 |
| LT | 5 | 2400 |
| CBD | 6 | 1608 |
| HA | 3 | 1368 |
| CWT | 3 | 1344 |
| MCCG | 6 | 1200 |
| YGH | 5 | 1200 |
| MCK | 4 | 1200 |
| LBK | 5 | 1200 |
| SJF | 2 | 960 |
| ASF | 2 | 840 |
| LM | 5 | 768 |
| **Mean** | **5.79** | **2166** |
| **Median** | **5** | **1272** |
| **Minimum** | **2** | **768** |
