# Supplementary material for: Somnotate: A probabilistic sleep stage classifier for studying vigilance state transitions
Source: PLoS Comput Biol. 2024 Jan 17;20(1):e1011793. doi: 10.1371/journal.pcbi.1011793 (PMC10824458; doi:10.1371/journal.pcbi.1011793)
Supplement: S2 Table — Manual and automated annotations of six 24 hour datasets were evaluated based on the consensus of multiple expert annotations. (DOCX) [file pcbi.1011793.s003.docx]

| **Dataset** | **Annotator** | **Method** | **Accuracy** | **Cohen's kappa** | **Weighted F1 score** |
| --- | --- | --- | --- | --- | --- |
| SampleFile_A | CBD | Manual | 0.952 | 0.916 | 0.952 |
| SampleFile_A | LEM | Manual | 0.936 | 0.887 | 0.936 |
| SampleFile_A | MCK | Manual | 0.934 | 0.884 | 0.934 |
| SampleFile_A | YGH | Manual | 0.878 | 0.785 | 0.876 |
| SampleFile_B | HA | Manual | 0.966 | 0.937 | 0.966 |
| SampleFile_B | LT | Manual | 0.957 | 0.92 | 0.956 |
| SampleFile_B | LM | Manual | 0.947 | 0.904 | 0.949 |
| SampleFile_B | TY | Manual | 0.97 | 0.945 | 0.97 |
| SampleFile_C | CWT | Manual | 0.957 | 0.925 | 0.957 |
| SampleFile_C | LBK | Manual | 0.957 | 0.926 | 0.958 |
| SampleFile_C | MCK | Manual | 0.962 | 0.934 | 0.962 |
| SampleFile_C | SJF | Manual | 0.952 | 0.915 | 0.949 |
| SampleFile_D | CBD | Manual | 0.948 | 0.908 | 0.946 |
| SampleFile_D | LBK | Manual | 0.933 | 0.884 | 0.934 |
| SampleFile_D | LM | Manual | 0.947 | 0.908 | 0.948 |
| SampleFile_D | HA | Manual | 0.954 | 0.921 | 0.955 |
| SampleFile_E | CWT | Manual | 0.961 | 0.931 | 0.961 |
| SampleFile_E | LEM | Manual | 0.967 | 0.941 | 0.967 |
| SampleFile_E | LT | Manual | 0.958 | 0.924 | 0.956 |
| SampleFile_E | MCK | Manual | 0.979 | 0.963 | 0.979 |
| SampleFile_F | HA | Manual | 0.956 | 0.923 | 0.956 |
| SampleFile_F | SJF | Manual | 0.941 | 0.897 | 0.942 |
| SampleFile_F | TY | Manual | 0.96 | 0.93 | 0.959 |
| SampleFile_F | VVV | Manual | 0.96 | 0.93 | 0.961 |
| SampleFile_F | YGH | Manual | 0.948 | 0.909 | 0.949 |
| SampleFile_A | Somnotate | Somnotate | 0.971 | 0.949 | 0.971 |
| SampleFile_B | Somnotate | Somnotate | 0.967 | 0.939 | 0.967 |
| SampleFile_C | Somnotate | Somnotate | 0.975 | 0.956 | 0.975 |
| SampleFile_D | Somnotate | Somnotate | 0.969 | 0.944 | 0.968 |
| SampleFile_E | Somnotate | Somnotate | 0.962 | 0.931 | 0.961 |
| SampleFile_F | Somnotate | Somnotate | 0.987 | 0.978 | 0.987 |
| SampleFile_A | SPINDLE | SPINDLE | 0.937 | 0.888 | 0.937 |
| SampleFile_B | SPINDLE | SPINDLE | 0.95 | 0.907 | 0.949 |
| SampleFile_C | SPINDLE | SPINDLE | 0.932 | 0.88 | 0.931 |
| SampleFile_D | SPINDLE | SPINDLE | 0.937 | 0.887 | 0.934 |
| SampleFile_E | SPINDLE | SPINDLE | 0.947 | 0.905 | 0.946 |
| SampleFile_F | SPINDLE | SPINDLE | 0.955 | 0.921 | 0.955 |
| SampleFile_A | IntelliSleepScorer | IntelliSleepScorer | 0.675 | 0.407 | 0.64 |
| SampleFile_B | IntelliSleepScorer | IntelliSleepScorer | 0.785 | 0.574 | 0.773 |
| SampleFile_C | IntelliSleepScorer | IntelliSleepScorer | 0.734 | 0.508 | 0.714 |
| SampleFile_D | IntelliSleepScorer | IntelliSleepScorer | 0.761 | 0.572 | 0.751 |
| SampleFile_E | IntelliSleepScorer | IntelliSleepScorer | 0.793 | 0.616 | 0.784 |
| SampleFile_F | IntelliSleepScorer | IntelliSleepScorer | 0.753 | 0.551 | 0.74 |
